# Method for analyzing HR-TEM micrographs to propose and/or describe structures and their interaction in crystalline materials

**DOI:** 10.1016/j.mex.2022.101855

**Published:** 2022-09-21

**Authors:** R. Obeso–Estrella, B. Pawelec, N. Mota, L.A. Flores–Sanchez, J.M. Quintana–Melgoza, R.I. Yocupicio–Gaxiola, T.A. Zepeda

**Affiliations:** a Universidad Autonoma de Baja California, Calzada Universidad 14418 Parque Industrial Internacional Tijuana, Baja California, Mexico C.P. 22390; b Instituto de Catálisis y Petroleoquímica, CSIC, c/Marie Curie 2, L10, Cantoblanco, 28049 Madrid, Spain; c Universidad Nacional Autónoma de México, Centro de Nanociencias y Nanotecnología, Ensenada, Baja California C.P. 22860, México; d Centro de Investigación Científica y de Educación Superior de Ensenada (CICESE), Ensenada, Baja California 22860, México

**Keywords:** Chemical composition, Structure models, Structural properties, Dynamics of structure growth, Crystalline structural characterization, Crystallographic planes measurements, Image processing of micrographs, Nonstoichiometric titanium oxides, Materials morphology

## Abstract

A general and versatile method for the analysis and processing of HR–TEM data useful for several applications is presented. The first utility is to identify the structures seen in the micrographs; also can be extended to propose the interaction of structure dynamics between various phases; and also it can be hybridized with the chemical method to make a proposal of new structure and/or phase. The general method consisted of four steps: 1) micrograph pretreatment, 2) measurement of planar distances, 3) structure identification, and 4) structure corroboration. Crystallographic planes were immediately identified by comparing the interplanar distances. Next, crystallographic data were collected from the Crystal Structures Database (ICSD) and introduced into Diamond software to visualize the planes in each structure. In addition, from the zone axis point of view it must show the planes aligned, similar as is observed in the HR–TEM micrograph.

• It was possible establish the growth mechanism of the different structures by identifying how is the structural interaction between the different oxides and sulfide phases.
• Method was successful applied to propose a new TiCoMoS sulfide phase through HR–TEM results.
• The method can also be extended to other areas where structural studies with HR–TEM are viable, such as biology, electronics, among others.

## Graphical abstract

**Figure:**
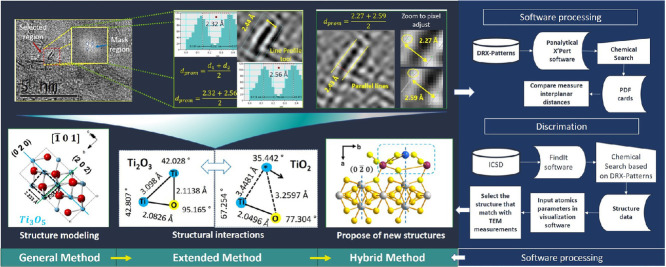

Specifications Table

**Table:** 

Subject Area:	Materials Science
More specific subject area:	Materials Chemistry
Method name:	Method for analyzing HR–TEM micrographs to propose and/or describe structures and their interaction in crystalline materials
Name and reference of original method:	N/A
Resource availability:	N/A

*Method details: A practical and efficient method for analyzing structural features from HR–TEM micrographs was developed and successfully applied to propose new structures and the structural model of the crystal growth mechanism, as well as to explain morphological changes in the materials.

## General Method

A general method was used to identify the structure and orientation of the phases detected in HR-TEM measurements. The steps of method included:

1) pretreatment of micrograph;
2) identify structure and phases;
3) discriminate the structure/phases;
4) corroborate the structure and
5) display structures.

All the steps performed are described bellow.

## Micrograph data treatment

### Micrograph Pretreatment

The original micrographs in .dm3 format were loaded in DigitalMicrograph™ 3.7.1 for GMS 1.2 Build 22 by Gatan software team. The images shown were duplicated from **Edit**>**Duplicate** image menu. When a region of interest was identify it was selected using the ROI tools rectangle. The size of rectangle was adjusted with right–clicking and holding down the “**Alt**” key to automatically adjust the ratio.

For the analysis of the region of the micrographs the Fourier Transform was applied and if considered necessary the mask was also applied; if not necessary only inverse Fourier Transform was applied to the same region obtaining a new image bounded in the region to perform a better analysis. Additional Laplacian filter could be applied in the case that the obtained image from FT filter was not clear yet from menu **Process**>**Spatial Filter**> **Laplacian or Process**>**Spatial Filter**> **Convolution**… and in the emergent windows select Type Laplacian of the list box then select in “Variant” the array of the filter (recommended prefer 3 × 3 1).

### Identify structure and phases

Planes were identify and measured realizing follow steps.

#### Measurement of interplanar distances

• Locate the points related to atoms on the micrographs.
• Create a line crossing towards the atoms.
• Use line tool localized in ROI tools to create the mentioned lines.
• Identify another equivalent plane (atoms parallel to the first one) and create other new line.
• Use line tool localized in ROI tools to create the mentioned lines.
• Measure the distance between these atoms
• From ROI tools select “line” and realize the measurements by placing the line between both two lines created (in the two previous steps). The line must be perpendicular to the two parallel lines created. Be careful with initial points of the lines used to measure the distances so that the start and end points are in position on the lines and do not cross them.
• Finally, capture the data displayed in “**Control**” tool as L.

Note: if distance between creation lines is not uniform (they are not parallel to each other) then the largest distance measured between atoms should be considered. It is important because from TEM micrograph a 2D image of a 3D object is obtained, so the tilt may result in an apparent deviation or distortion of observed atoms and planes.

#### Planes identification

To identification of the planes was realized a comparative analysis, which is, described below.

In **X´Pert HighScore Plus** version 2.2a (2.2.1) software from menu **Reference patterns**>**Retieve_Patters_by**>**Restrictions…**

In the tab **Chemistry** click in **Periodic Table…** then select the element taking into account the composition of the sample; for example, Al, Ti and O to the case of Al_2_O_3_–TiO_x_ materials.

Next click in “**OK**” and after click in “**Add Rest to None of**” to finally click in “**Load**”. In the tab “**Pattern List**” view each pattern data and compare them with the measured interplanar distances.

Note: as criterion for accepting interplanar distances as correct, the allowed variation was considerate to be ±0.1Å. If several phases show similar interplanar distances, each of them should be considerate as possible phase referred to the analyzed TEM micrograph region.

From this comparative analysis, the planes related to measured interplanar distances are obtained.

### Discriminate phases/structures to elucidate the presented in TEM results

In this step, we can obtain the necessary data to establish a structural model as well as discriminate between several possible structures if required. Therefore, if after applying all the previous steps several structures (two or more) have been obtained as possible or candidate structures, then this step is necessary to discriminate between them.

#### Identification of possible phases

Perform a new search for the identified phases as possible using FindIt software version 1.9.3 with the Crystal Structure Database (ICSD) version 2013-2 by Fachinformationszentrum Karlsruhe, Germany. In addition, search can be done also in COD (crystallographic open database).

From the “**Search ICSD**” window with the “**Chemistry**” tab active in the “**Type**” section check the “**Exclusive AND**” box and then select the atoms of the elements that make up the material; e.g. Aluminum (Al), Titanium (Ti) and oxygen (O) for Al_2_O_3_-TiO_x_ materials. Then click on the “**Search**” button.

#### Element selection

Select each element that has a distinct structure or phase for proper discrimination. You can save each element as an ASCII.CSV file from the main Windows menu **File** > **Export Current Length** to obtain the atomic position data for each possible structure and/or phase identified.

#### Visualize the previously identified planes

Load the crystallographic data into the visualization software (Diamond version 3.0e) using the menu **Structure** > **Atomic Parameters...** and load the atomic positions (wyckoff sites), coordinates (x, y, z), oxidation numbers and Site Occupancy Factor (S.O.F.).

#### Phase/structure selection

The following were considered for the phase/structure selection criteria.

##### Visually corroborate the same zonal axis in each of the visualized planes

The visualized structure was rotated along x, y and z axis. This to achieve a view where each visualized plane is observed as lines. This visually corroborates the same zonal axis to each of the displayed planes. If it is possible to find a view as above (where all planes have the same zone axis); all structures/phases that comply with this should be considered as structure/phase candidates. Similarly, all other structures/phases that do not meet the above criteria should be discarded.

##### Contrast the obtained visualization (of the zone axis) with the experimental region of the TEM micrograph

The criteria to confirm the visualized structure belong to the analyzed micrograph. Visually the planes must be with the same orientation. At this point, the angles between the planes, which must be similar, are visually confirmed. Only a small deviation can be tolerated (±7.5 % referent to inclination of the planes in grades respect to x, y and z axis) taking into account the possible variation related to the 2D projection of the micrograph with respect to the structure/phase visualized in the software.

### Confirm the structure

If the above steps are insufficient to determine a single structure/phase or if it is desired to confirm the result, a simulation of that structure can be performed to discriminate or confirm, depending on the case.

To corroborate the structure you must have a file with extension .xyz of the structure in question. If you do not have this file you can generate it using the visualization software (Diamond version 3.0e) by saving the file with extension .xyz of the visualized structure from the menu

**File** > **Save as** > **Save structure as...** and in the dialog box select as the format to save the XYZ file type with extension .xyz.

Note: Also can use files with PDB (Protein Data Bank) format.

Open the .xyz or .pdb file with a TEM simulator (SimulaTEM by Alfredo Gómez Rodríguez and Luis Manuel Beltrán Del Río, 2010) [1]. Click on “**Load file**” and browse to the address where your file is located and open it.

Introduce the condition to simulate the TEM micrograph. In this step the **I, J** and **K** coordinates for the indexed alignment of the beam direction are imported by clicking on the “**Rotation**” tab and entering the **I, J** and **K** values and clicking on the “**Align**” button. The **I, J** and **K** values are the same as the zonal axis obtained in the Diamond viewing software version 3.0e (from the **Picture** > **Viewing Direction...** menu) by taking the “**Current settings for hkl =**” data located in the “**View towards plane**” section of the dialog box.

It should be rotated to visually achieve the image similar to the experimental micrograph. Various settings such as microscopy and illumination parameters can also be varied. It is recommended to obtain the images from the Focus series for comparative visual analysis. To do this just click on the **Focus** tab and the opportunity settings are desired and click on the “**Go**” tab to obtain the series of the images by varying the focus.

Change the settings to obtain a suitable visualization and an image similar to the experimental one to corroborate or discard the simulated structure or phase.

## Extended Method

The Extended Method of the General Method was used for the analysis of the structural growth mechanisms by identifying the atom connections between the crystallographic structures previously identified. The following steps were carried out to achieve this objective:

1. To apply the Extended Method is required previously identify two vicinal phases/structures using the General Method.
2. The data of both vicinal structures should be compared to identify three atoms with similar distances and angles between them (p. eg. see Figure 3).
a. To identify three atoms with similar distances and angles between them should check the distances between atoms in each vicinal structure. This step can be done visually or comparing the distance and angles data between atoms. This information can display from the menu **View** > **Table** > **Distances** and **View** > **Table** > **Angles** in Diamond software (version 3.0e).

Note: If are found more than one group of three atoms with similar distances and angles then several ways of connecting both vicinal structures are possible. An example on this can see in Figure 3 to Ti_2_O_3_ trigonal phase, such three distinct triad of atoms were identified, then three different ways to connecting with other structure (TiO_2_ anatase tetragonal) are possible.

3. Compare modeled structures with experimental micrograph considering the orientation of structure/phase (zone axis). It could help to discriminate which atoms really are involved in structural connections. Figure 4 shows an example of obtained rotated model structure considering the TEM image from experiment.
4. Identify the crystallographic planes by which the structures are connecting between them.
  a. Select three previous identified atoms.
    i. Click in the atoms (three) maintaining pressed the “**Ctrl”** Key to select every three atoms.
  b. Generate plane that passes through the three atoms.
    i. From menu **Object** > **Planes** > **Create Plane Through Atoms**… and click in “**Accept**”.
5. Represent the connection between both vicinal structures.
  a. In visualization software, found the best view to perceive the connected atoms.
  b. Additionally, can extract a representation of atoms that are connected. (Figure 3 shows an example).
6. Hence, the growth mechanisms were realized based on the obtained information.

## Hybrid Method

A hybrid method consisting of a complementary structural and chemical analysis has been used to suggest a new structure of the metallic sulfide phase. After each of the previous steps (application of the General Method and the Extended Method), additionally, the structural proposal must be corroborated from the chemical point of view. To do so, a chemical simulation was performed.

1. Construct or draw the structure in a chemical software PerkinElmer ChemDraw® version 17.0.0.206 based on the structure obtained in the Diamon 3.0e visualization software from the application of the above methods.
2. Get the 3D viewer.
  a. Select the structure.
  b. Navigate to **Edit** > **Get 3D Model**. The 3D structure appears in the work area of document (ChemDraw windows).
  c. Double click on new 3D representation to open **Chem3D windows**. Then the 3D structure will displayed.
3. To relaxing the chemical structure was applied molecular dynamics simulation by Molecular Mechanics method (MM2) for locating stable conformations. Hence, MM2 method to minimize the energy from the menu **Calculations** > **MM2** > **Minimize Energy**… and then click in “**Run**”.
4. Finally, the chemical structure obtained must be compared with the one proposed in the Diamond 3.0e software.

## Method validation: Application of the Method in a case of study

General Method was successful to identify several titanium oxides and sulfide structures/phases contained in CoMo sulfide catalysts supported on gamma alumina–titania mixed oxides [2]. On this way, applying the General and Extended Method was possible identify the orthorhombic TiO_2_ phase and an explication about how TiO_2_ particles interact between them to form structures of nanorods morphology was modeled [3].

## General Method: Identify and building of structure/phases models

**Fig. 1:**
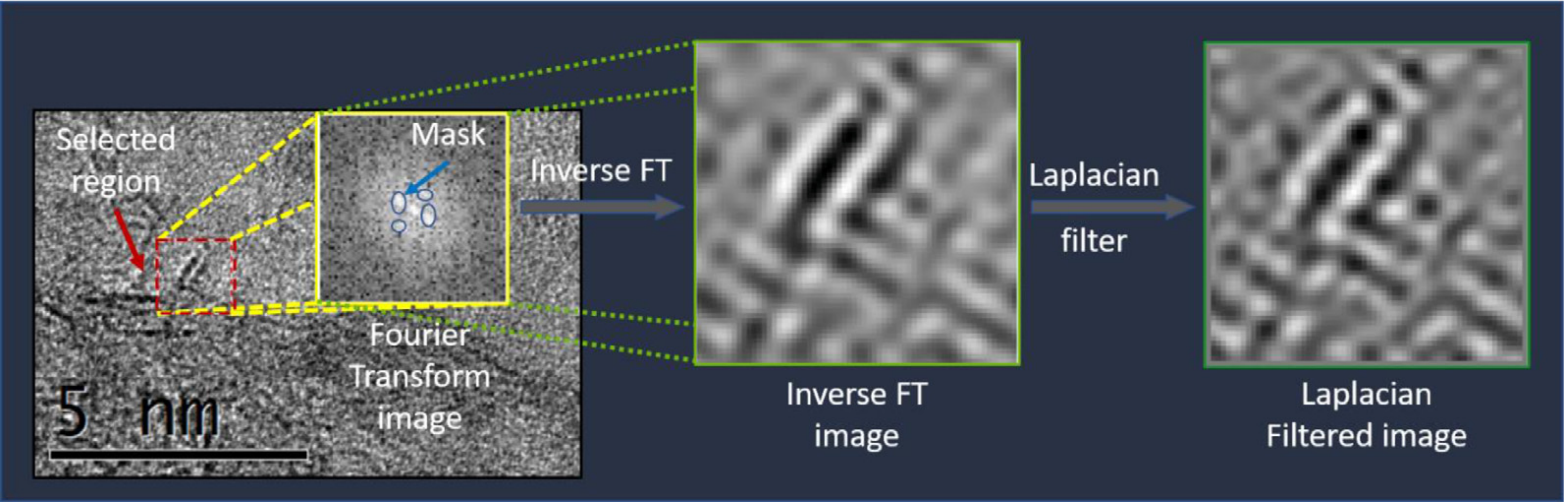
Process of micrograph filtering pretreatment of a selected region for analysis.

Region of a micrograph was selected and Fourier Transform (FT) filter was applied with an oval mask. The ovals were adjusted to the equivalent diffraction points displayed in FT image. Hence, inverse transform was applied and new filtered image were obtained. Additional Laplacian filter could be applied in the case that the obtained image from FT filter was not clear yet. This new filtered image was performed the measurement of interplanar distances (Figure 1).

**Fig. 2:**
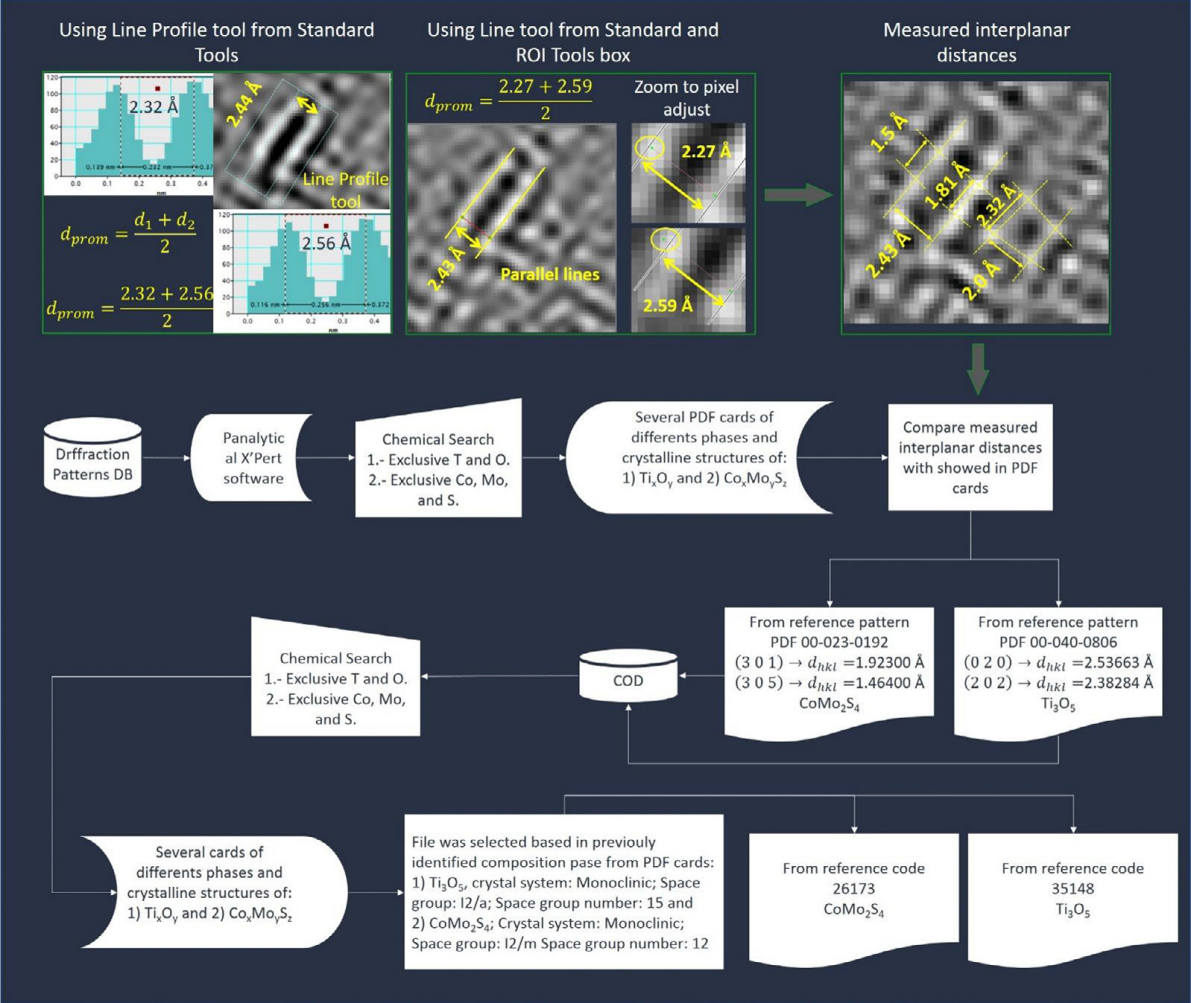
Process to discriminate the structure based on data measured and extracted from data base.

**Fig. 3:**
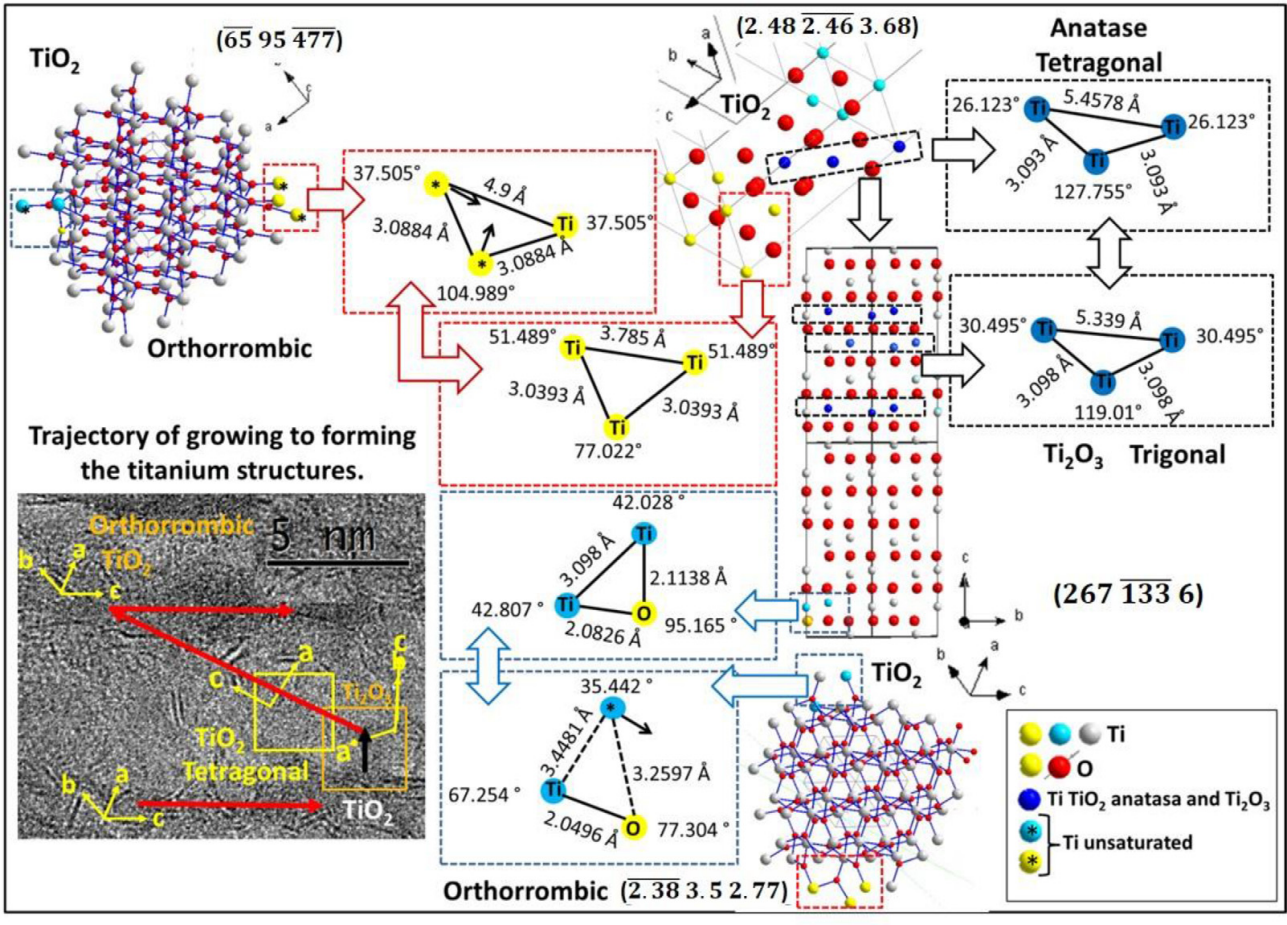
Schematic of several simulated titanium oxide structures applying the general method and their connections (by extended method) to elucidate the grown mechanism of visualized structure in HR–TEM micrograph.

**Fig. 4:**
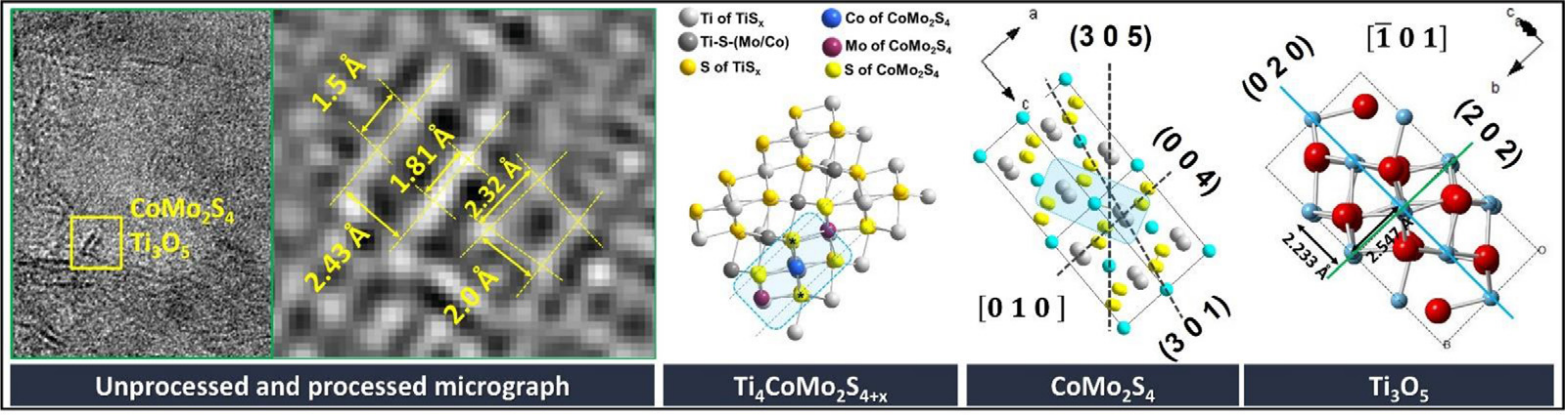
Shows CoMo_2_S_4_ phase detected over Ti_3_O_5_ structure from CoMo–12 micrograph (Unprocessed micrograph). Fourier transform images for CoMo_2_S_4_ and Ti_3_O_5_ structures also are showed (processed micrograph). Further, simulated structures from PDF 00-023-0192 and PDF 40-0806 for each structure are displayed on the right.

Subsequently, interplanar distances were compare with obtained distances extracted from ICSD database and a discrimination process was performed (Figure 2). Thus, several structures could be simulated applying the general method (Figures 3 and 4).

## Extended method: interaction between structures to propose a mechanism of growing of several structures of titanium oxides with different morphologies

Stoichiometric and nonstoichiometric titanium oxide structures were identified in HR–TEM results, and then Extended Method was applied to known possible connection between every detected structures and was proposed to elucidate the grown mechanism (Figure 3).

## Hybrid Method

Nonstoichiometric titanium oxide type Ti_2_O_3_ and a layer CoMo_2_S_4_ vicinal structure were identified in HR–TEM results, then Extended Method was applied to known possible connection between both structures [2]. In the same way, also was detected others vicinal structures; Ti_3_O_5_ and a layer CoMo_2_S_4_, however in this case the resulting structure did not founded by discrimination as was mentioned in General Method, i. e. none PDF card from ICSD data base could be related to structure proposed which should have titanium (Ti), cobalt (Co), molybdenum (Mo) and sulfur (S) atoms in its composition.

Therefore, must be considerate the interaction between CoMo_2_S_4_ sulfide and Ti_3_O_5_ oxide to create a new phase/structure. Moreover, propose should be based in suitable structural and chemical interaction to write in related references [4,5].

Hence, through of both structures (from PDF 00-023-0192 to and PDF 40-0806 to structure are displayed) was propose a new interaction structure. Applying the Extended method found the three atoms with similar distances and angles in both visualized Ti_3_O_5_ and CoMo_2_S_4_ structures (Figure 4).

**Fig. 5:**
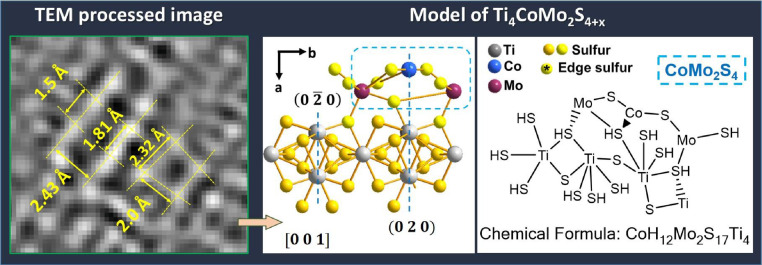
Schematic views of TiCoMoS (Ti_4_CoMo_2_S_4+x_) model based from observations from HR–TEM observations (left image): structural model from extended method using Diamond (centered image) and chemical model obtained with Chemdraw (right) software.

To confirm this structural model, an additional analysis has been performed. Then, a chemical analysis was realized which consisted in draw the structure in chemical visualization software (Chemdraw) (Figure 5). Later, this chemical structure was convert to a 3D molecule and after was subjected to a relaxing of its chemical structure applying Molecular Method (MM2) to reach the minimal energy relaxing structure. This relaxing molecule was contrasted with that structural model obtained from extended method to corroborate the viability of that chemical structure propose. This because Diamond software permits structural modeled whereas ChemDraw software was used to model the chemical molecule. Then, based on the results where the similar 3D-structures were obtained from Diamond and ChemDraw models (Figure 5 left and centered image), we could confirm that the proposed phase is viable by both as the structural as well as chemical viewpoint.

**Fig. 6:**
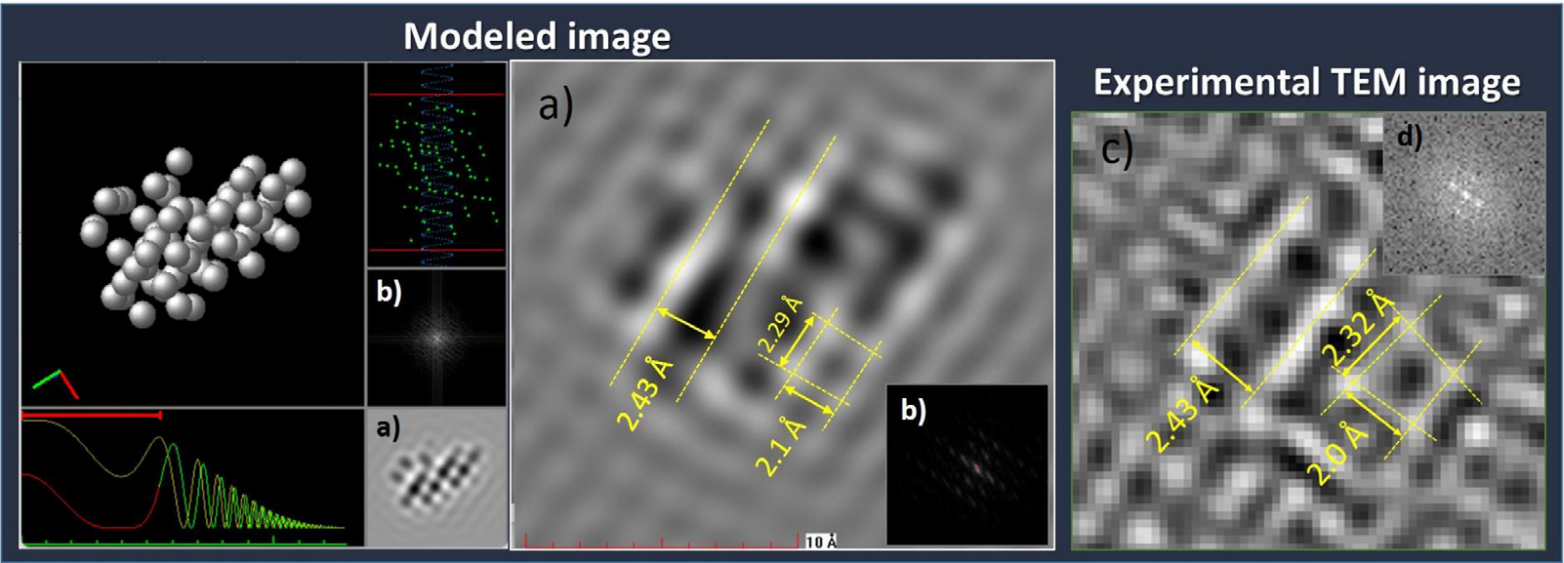
Image obtained from SimulaTEM software modeling: a) TEM image and its b) Fourier Transform. Incise c) and d) corresponding to TEM image and Fourier transform obtained from experimental analysis, respectively.

Note: In addition, from SimulaTEM simulation is obtained the Fourier Transform image which also can compare with experimental image (Figure 6 b) and d)).

Additionally, the structure of Ti_4_CoMoS_4+x_ also was confirmed by modeling of TEM image using SimulaTEM software. First, the data of structure Ti_4_CoMoS_4+x_ (centered image in Figure 5) was save it as .pdb file. Next, this file was loaded in SimulaTEM software and the displayed structure was rotated until result an image similar to that visualized from TEM experiments. Finally, the interplanar distances were measured in experimental as well as simulated images to confirm the proposed structure (Figure 6 a) and c)).

## Declaration of Competing Interest

The authors declare that they have no known competing financial interests or personal relationships that could have appeared to influence the work reported in this paper.

## Data Availability

The authors are unable or have chosen not to specify which data has been used.

## Appendix Supplementary materials

Image, image 1

Image, image 2

Image, image 3

Image, image 4
