# Supplementary figures and images for: Method for analyzing HR-TEM micrographs to propose and/or describe structures and their interaction in crystalline materials
Source: MethodsX. 2022 Sep 21;9:101855. doi: 10.1016/j.mex.2022.101855 (PMC9523379; doi:10.1016/j.mex.2022.101855)

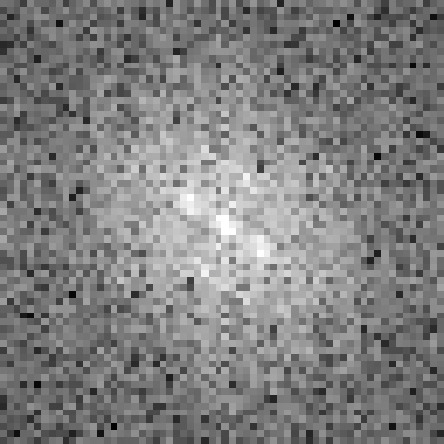

Supplement: Supplementary file 1 [file mmc1.jpg]

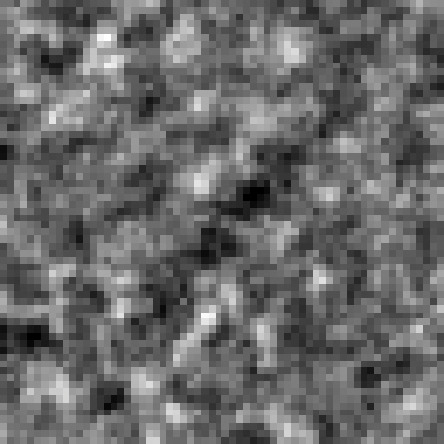

Supplement: Supplementary file 2 [file mmc2.jpg]

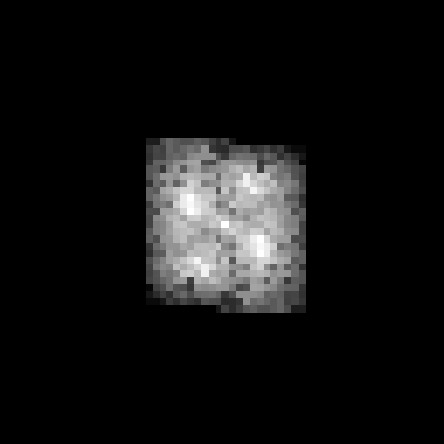

Supplement: Supplementary file 3 [file mmc3.jpg]

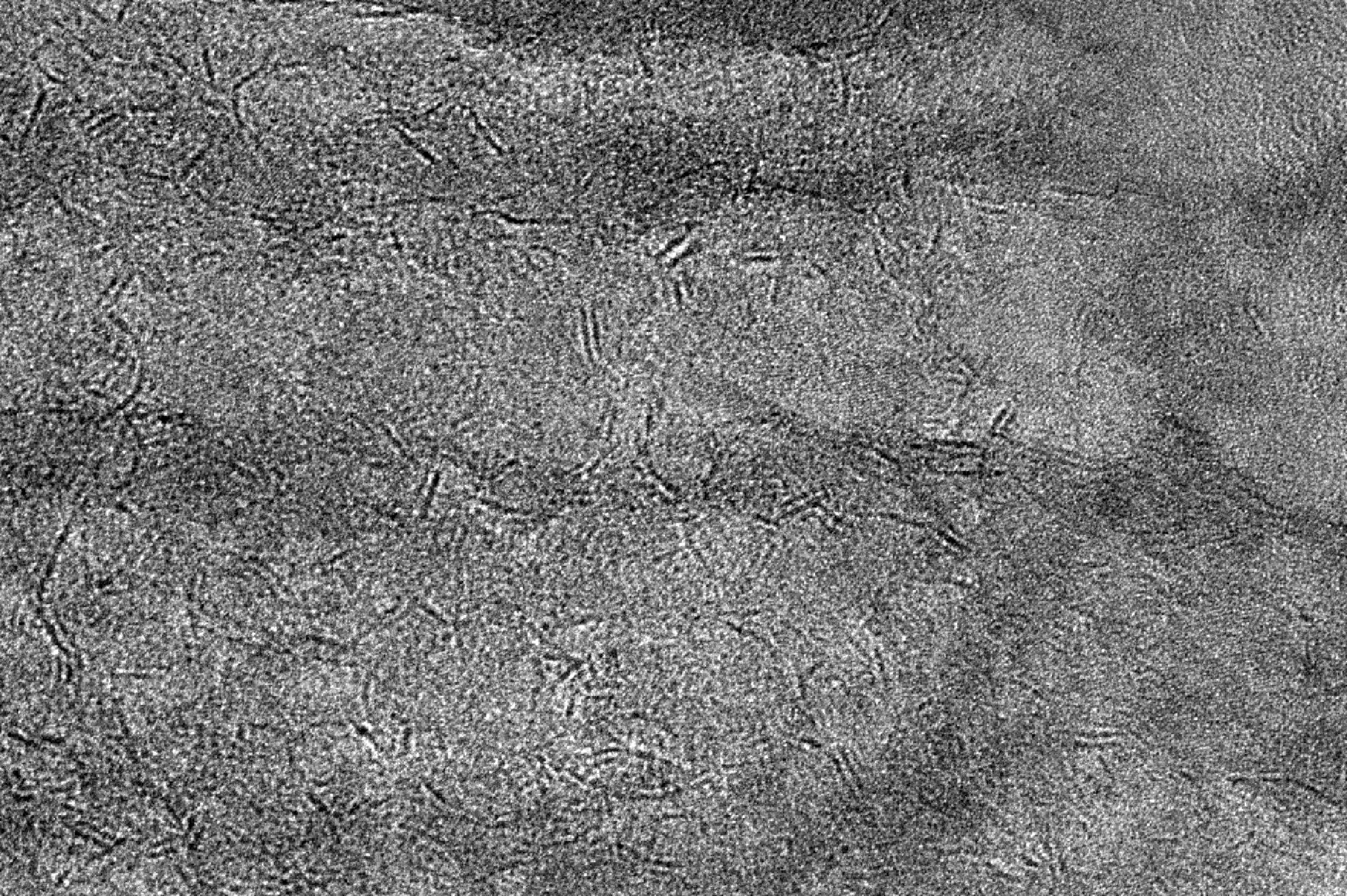

Supplement: Supplementary file 4 [file mmc4.jpg]
